# Pilot Study of a Web-Delivered Multicomponent Intervention for Rural Teens with Poorly Controlled Type 1 Diabetes

**DOI:** 10.1155/2016/7485613

**Published:** 2016-08-17

**Authors:** Amy Hughes Lansing, Catherine Stanger, Alan Budney, Ann S. Christiano, Samuel J. Casella

**Affiliations:** ^1^Department of Psychiatry, Geisel School of Medicine at Dartmouth, Lebanon, NH 03766, USA; ^2^Children's Hospital at Dartmouth, Dartmouth-Hitchcock Medical Center, Lebanon, NH 03766, USA

## Abstract

*Objective*. The purpose of this study was to examine the feasibility and effectiveness of a web-delivered multicomponent behavioral and family-based intervention targeting self-regulation and self-monitoring of blood glucose levels (SMBG) and glycemic control (HbA1c) in teens with type 1 diabetes (T1DM) living in rural US.* Methods*. 15 teens with poorly controlled T1DM participated in a 25-week web-delivered intervention with two phases, active treatment (weekly treatment sessions and working memory training program) and maintenance treatment (fading of treatment sessions).* Results*. Almost all (13 of 15) participants completed at least 14 of 15 treatment sessions and at least 20 of 25 working memory training sessions. SMBG was increased significantly at end of active and maintenance treatment, and HbA1c was decreased at end of active treatment (*p*'s ≤ 0.05). Executive functioning improved at end of maintenance treatment: performance on working memory and inhibitory control tasks significantly improved (*p*'s ≤ 0.02) and parents reported fewer problems with executive functioning (*p* = 0.05). Improvement in inhibitory control was correlated with increases in SMBG and decreases in HbA1c.* Conclusions*. An innovative web-delivered and multicomponent intervention was feasible for teens with poorly controlled T1DM and their families living in rural US and associated with significant improvements in SMBG and HbA1c.

## 1. Introduction

Management of type 1 diabetes involves the completion of multiple daily adherence behaviors that may be complex and often disruptive to daily life (e.g., blood glucose checking at least four times per day, correctly calculating, and administering insulin doses). Adolescents with type 1 diabetes often struggle to maintain adherence to the recommended frequency of self-monitoring of blood glucose (SMBG) and achieve optimal metabolic control (HbA1c). Interventions targeting adherence in youth with type 1 diabetes, including those with coping skills, motivational, cognitive behavioral, and family systems components, have typically shown only small to moderate effect size improvements in adherence behaviors and HbA1c [1, 2]. Research has suggested that deficits in self-regulation and underlying executive function are associated with poorer adherence and higher HbA1c in teens with type 1 diabetes [3, 4]. The development of interventions that specifically target self-regulation and executive function may be particularly beneficial for youth with type 1 diabetes [5]. Further, among studied interventions targeting adherence, none have specifically targeted a rural population and only one has been translated into a web-delivered format, behavioral family systems therapy for diabetes [6, 7]. Thus, there is a need to develop more effective behavioral interventions for nonadherence and, in particular, interventions that can be delivered to families in rural regions with limited access to specialized behavioral health and pediatric endocrinology services [8].

This paper describes a pilot study of a multicomponent intervention that integrated a behavioral and family-based treatment [9] with a working memory training program to promote youths' developing self-regulation, encourage optimal diabetes management, and improve glycemic control. Specifically, this intervention provided (1) behavior economic incentives to teens for daily self-monitoring of blood glucose (SMBG) and to parents for parental monitoring of SMBG and (2) cognitive behavioral and motivational therapy [9, 10], along with (3) a working memory training program for teens [11]. The intervention included both an active treatment (ActiveTX) phase where incentives and therapy were delivered weekly along with the working memory training program, as well as a maintenance treatment phase (MaintTX) where incentives and therapy were faded, with an increasing length of time between sessions.

This intervention was based on a previously piloted behavioral and family-based intervention for teens with poorly controlled type 1 diabetes [9]; however, this prior study did not include a maintenance phase or working memory training and was delivered through face-to-face sessions in a metropolitan region in southern US. In this previous pilot study, an inclusion criterion for participation was living within 30 miles of the treatment center to ensure that families could reasonably and regularly attend the weekly therapy sessions. In translating this intervention model for delivery in a more rural region of the US, it was necessary to implement the intervention without using a clinic-based model. Web-delivery of the entire intervention, including both the treatment sessions and working memory training program, was selected as broadband Internet penetration in the region was around 75% of households and expected to increase during the course of the trial. The distance many families lived from the pediatric endocrinology clinic and the treatment intensity (3 months of weekly sessions and then 3 months of fading treatment sessions) made a home-based visit model incompatible with providing a time and cost-effective treatment.

The primary components of this multicomponent intervention were selected for efficacy in targeting self-regulation of health behaviors in teens. Behavior economic intervention approaches are promising new methods for improving self-regulation of health behaviors. Behavioral economic incentives (BEI) are grounded in neuroeconomic theory [12], which purports that self-regulation failure involves overly valuing immediate rewards and devaluing future rewards. There is an expanding literature on incentive use to increase a wide range of health behaviors [13] such as increasing SMBG among teens with type 1 diabetes [9, 14] and losing weight [15]. Interventions like BEI that reward the initiation of healthy behaviors that lack immediate inherent reward, for example, SMBG, may improve self-regulation and facilitate the development of more habitual completion of healthy behaviors. Further, adolescence may be an ideal time to use incentives to improve adherence. One study has shown that the use of rewards facilitates self-regulation among adolescents to a greater extent than for adults [16], suggesting that adolescents' neural functions might be more influenced by immediate rewards than adults. Thus, incentivizing SMBG, an often unpleasant and inherently unrewarding health behavior may be an important tool for improving self-regulation as well as increasing SMBG and in turn improving HbA1c in youth with poorly controlled type 1 diabetes.

This intervention model also provided behavior economic incentives to parents towards increasing parental monitoring of adolescents' SMBG behaviors. Parents are critical to the development of adolescent self-regulation, and in particular, parental monitoring has been identified as a key predictor of adherence and glycemic control in youth with type 1 diabetes [17–19]. Incentivizing parental monitoring and parental implementation of behavioral contracts for youth SMBG may help to establish family environments that are more supportive of the development of youth self-regulatory skills for diabetes management. Such family environments would be more conducive to effective parent-child collaboration in diabetes management that is seen in families of adolescents with more optimal glycemic control [17, 20].

Cognitive training interventions, in particular working memory training, are associated with improvements in executive functioning in youth and may also benefit teens with type 1 diabetes. Working memory is a cognitive system that actively holds information in the mind permitting verbal and nonverbal activities such as reasoning and comprehension processing [21]. Working memory is an executive function that involves goal-oriented active monitoring or manipulation of information. Further, performance deficits on inhibitory control and risky decision-making tasks are also related to working memory capacity [22]. Studies show that executive function deficits, including those related to working memory, are associated with adherence and metabolic control in youth with type 1 diabetes [3, 23].

Working memory training involves practice of increasingly difficult verbal and visuospatial tasks requiring the temporary storage and manipulation of information. Working memory training aims to improve executive function and decision-making by strengthening working memory neurocognitive processes through practice. There is also increasing evidence demonstrating that commercially available computerized working memory training programs can reliably enhance executive function in youth with ADHD, teens with extremely low birth weight history, and teens with moderate cognitive deficits [24–26]. Working memory training not only improves working memory performance, but has also been found to enhance performance on other cognitive tasks that have not been trained including decision-making [27]. Thus, working memory training may be a useful tool for improving executive function and adherence in youth with poorly controlled type 1 diabetes and was integrated into this pilot intervention model.

A primary aim of this pilot study was to examine the feasibility of providing a web-delivered multicomponent intervention in a rural region of the US. Frequency of exclusion due to lack of broadband Internet as well as treatment completion rates is explored. With regard to our secondary aim to examine treatment efficacy, we hypothesized that (1) teen and parent participation in the intervention would increase teen SMBG and decrease HbA1c and that changes in SMBG and HbA1c at the end of ActiveTX would be maintained at the end of MaintTX, (2) participation in the intervention would improve teen executive functioning on both objective behavioral and parent report measures, and (3) changes in executive functioning would be associated with changes in SMBG and HbA1c.

## 2. Methods

Dartmouth College's Institutional Review Board approved the study. Fifteen teens (47% female, 60% using an insulin pump, average age = 15.8 years, range = 13.6–17.5 years, average length of diagnosis = 6.4 years) with poorly controlled type 1 diabetes were recruited from a pediatric type 1 diabetes clinic. 12 of 15 adolescents lived in Health Resources and Services Administration (HRSA) defined rural regions. Although the 3 remaining participants lived just outside of a HRSA defined rural region, they each travelled at least 90 miles to reach their pediatric endocrinology clinic. Inclusion criteria included age 13–17, average HbA1c ≥ 64 mmol/mol (8%) for past 6 months, most recent HbA1c ≥ 64 mmol/mol (8%), type 1 diabetes duration >18 months, at least one parent/guardian participant, and a computer with broadband Internet at home. Exclusion criteria included pregnancy and severe medical or psychiatric illness. All teens were provided with a meter and testing strips during the course of their participation in the study. There were 9 teens screened for the program that were eligible but declined to participate due to lack of interest or being too busy at the time. There was not a significant difference in HbA1c at the date of screening between those teens that participated and those that declined (*t*(22) = −0.73,* p* = 0.47). Additionally, there were 3 teens that were otherwise eligible for the study but were excluded due to not having a computer with broadband Internet at home. Families were loaned a web camera if they did not have working cameras on their PCs or laptops. Intake and follow-up assessments were conducted in person at the endocrinology clinic.

### 2.1. Intervention

#### 2.1.1. Active Treatment (ActiveTX)

The 11-week ActiveTX included weekly behavior economic incentives (BEI), brief motivational enhancement and cognitive behavioral therapy (MET/CBT) sessions, and working memory training (WMT) all delivered over the Internet.

For teens,* BEI* involved a 2-week baseline phase when teens received $10 per week for uploading their blood glucose meters to a personal blood glucose data management website, Carelink. From week 3 to week 11, weekly incentives were earned for meeting the SMBG goal, testing ≥5 times daily (>2 hours apart), on an increasing number of days per week. Incentives escalated from $10 to a maximum of $30 and a weekly $5 bonus for exceeding the number of days meeting the goal. In week 3, the initial target for days meeting the SMBG goal to earn incentive was individualized, set at one day more than achieved during week 2. During weeks 4–7, the target number of days increased by 1 if the prior target was met, up to 5 days per week, and then in weeks 8–11 the target was set at 5 days per week for all participants.

Parents also participated in* BEI*. Weekly web-delivered sessions were used to develop and implement a home SMBG contract specifying small daily rewards and consequences for teens meeting SMBG goals. To encourage parent monitoring and implementation of the contract, parents earned incentives for providing daily reports to the clinic. Parent reporting goals were always set at 5 days per week, with the same escalating earning system, bonuses, and dollar amounts as teens.

In weeks 1–11, teens also received weekly 20-minute web-delivered* MET/CBT* sessions, which coincided with awarding of incentives, focused on improving SMBG and other self-care behaviors using motivational interviewing and cognitive behavioral principles.

Beginning in week 3 of ActiveTx, teens completed* WMT*, via Cogmed-RM v.2 [11]. Teens were to complete 25 WMT sessions during active treatment, 5 per week optimally. Each session lasted about 1 hour and included 8 different training tasks and then a game could be played at the end of the session. Youth could earn up to $10 for each WMT session completed. This included $5 for completing the session in a single day and a $5 bonus for good performance in the session, indexed as improving or maintaining performance on 3 out of 8 training tasks. Weekly coaching calls from research staff provided feedback and motivational support to teens and parents to facilitate continued improvements and completion of sessions.

#### 2.1.2. Maintenance Treatment (MaintTX)

The 14-week MaintTX included fading of BEI and MET/CBT sessions. Incentives were awarded and MET/CBT sessions occurred only on weeks 13, 16, 20, and 25. The weekly BEI reward magnitude remained the same ($30 per week, $5 bonus). To encourage weekly family review of SMBG, teens and parents earned $5 per weekly upload.

Across ActiveTX and MaintTX, the maximum BEI earnings for teens and parents were $845 each. The teen could earn an additional $245 from WMT. Incentives were remotely loaded onto a study-provided debit card. Also, families were encouraged, but not required, to contact their diabetes educator. Educators also communicated back to the counselor if there were specific concerns and/or goals for individual patients.  Table 1 provides an overview of the treatment model.

### 2.2. Measures

#### 2.2.1. SMBG

To assess SMBG frequency, the total number of SMBGs a day during the 14 days prior to each assessment point was recorded to calculate an average daily frequency. Blood glucose data were gathered from blood glucose meters. SMBG was assessed PreTX, at the end of ActiveTX, and at the end of MaintTX.

#### 2.2.2. HbA1c

HbA1c was assessed during endocrinology clinic and study assessment visits. HbA1c was assessed PreTX, at the end of ActiveTX, and at the end of MaintTX.

#### 2.2.3. Executive Functioning

Executive functioning was assessed pretreatment (PreTX) and at the end of MaintTX, but not at the end of ActiveTX. Measures to assess executive functioning were selected to capture changes in working memory and related changes in inhibitory control as well as more global parent reports of executive functioning. To assess working memory, the* digit span* subtest of the Wechsler Adult Intelligence Scale (WAIS-IV) [28], for youth age 16 or older, or the Wechsler Intelligence Scale for Children (WISC-IV) [29], for youth younger than 16, was administered. The scaled score from the digit span subtest was utilized, where higher scores indicated better working memory capacity. To assess* inhibitory control*, the Delis-Kaplan Executive Function System [30], color word interference subtest was administered. Specifically, the Condition 3 Inhibition Completion Time Scaled Score and Errors Scaled Score were utilized, where higher scores indicated better inhibitory control. Parents completed the Behavior Rating Inventory of Executive Function [31] to provide ratings of teen's* executive functioning in everyday life*. The Global Executive Composite (GEC) *T*-Score was utilized, with higher scores indicating greater problems in executive functioning.

### 2.3. Statistical Analyses

Change in SMBG, HbA1c, and executive functioning were assessed through paired* t* tests. Cohen's* d* effect sizes were also calculated. For SMBG and HbA1c changes from PreTX to ActiveTX, PreTX to MaintTX, and ActiveTX to MaintTX were assessed. Executive functioning only included measures at PreTX and MaintTX. Analyses to examine associations between changes in executive functioning, SMBG, and HbA1c utilized PreTX to MaintTX change scores that were calculated for each measure. Pearson correlations were used to examine associations between change scores. Participants were included in all analyses regardless of the number of weeks of treatment completed. At the end of ActiveTX all 15 participants had HbA1c measured, while 1 participant did not provide SMBG data. At the end of MaintTX all 15 participants had HbAlc measured and all 15 provided SMBG data. One participant did not complete the executive functioning assessment at the end of MaintTX.

## 3. Results

### 3.1. Treatment Adherence and Incentive Earnings

Almost all (13 of 15) participants completed at least 14 out of 15 of the BEI + MET/CBT treatment sessions. For WMT, 14 of 15 participants completed at least 20 of 25 sessions. Youth trained an average of 3.71 times per week (SD = 1.23) and improved an average of 60% of tasks relative to prior performance on the same task. On average, teens earned $419 from BEI and $181 from WMT, while parents earned $445.

### 3.2. Self-Monitoring of Blood Glucose

**Figure 1:**
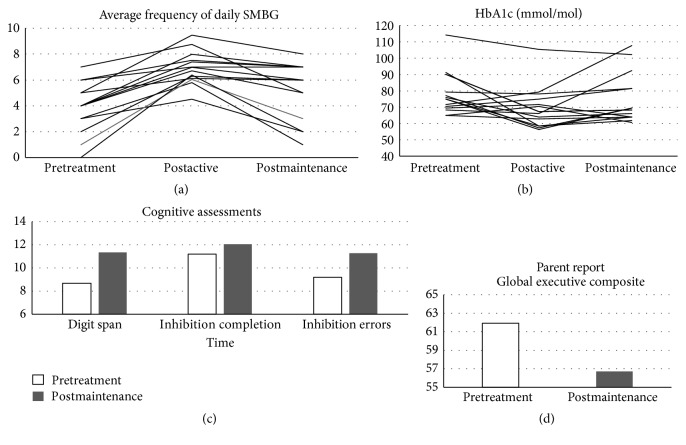
Individual participants' frequency of self-monitoring of blood glucose (SMBG (a)), glycosylated hemoglobin levels (HbA1c (b)) and average scores for all participants on cognitive functioning assessment and scaled scores (c) and *T*-scores (d).

Figure 1(a) shows SMBG data for each participant. Compared to PreTX (3.73 ± 1.70), SMBG was significantly increased at end of ActiveTX (6.92 ± 1.26; *t*(13) = −7.78,* p* < 0.001,* d* = 1.56), and the effect size was large. SMBG was also significantly increased compared to PreTX at the end of MaintTX (4.87 ± 2.45; *t*(14) = −2.19,* p* = 0.05,* d* = 0.54), with a medium effect size. Although significantly higher than at PreTX, SMBG at end of MaintTX was significantly decreased from the end of ActiveTX (*t*(13) = 4.00,* p* = 0.002).

### 3.3. Glycemic Control

Figure 1(b) shows HbA1c data for each participant. Compared to PreTX (80 ± 15 mmol/mol (9.5 ± 1.4%)), HbA1c was significantly decreased at end of ActiveTX (71 ± 15 mmol/mol (8.7 ± 1.4%); *t*(14) = 2.74,* p* = 0.02.* d* = 0.62), with a medium effect size. Six participants achieved >11 mmol/mol (1%) decrease in HbA1c at end of ActiveTX. HbA1c was not significantly decreased at end of MaintTX (76 ± 15 mmol/mol (9.1 ± 1.4%); *t*(14) = 0.89,* p* = 0.39,* d* = 0.29). Of note, 11 of 15 teens did maintain a lower HbA1c at end of MaintTX compared to PreTx, and 5 participants maintained >11 mmol/mol (1%) decrease in HbA1c at the end of MaintTX. HbA1c at the end of MaintTX did not significantly differ from HbA1c the end of ActiveTX (*t*(14) = −1.43,* p* = 0.17).

### 3.4. Executive Functioning

Participants improved on performance tasks measuring working memory and inhibitory control and parents reported fewer problems with executive functioning at the end of MaintTX.  Figure 1(c) shows average scores on executive functioning task assessments. Teens improved on digit span scaled scores (PreTX = 8.60 ± 2.41; MaintTX = 11.36 ± 3.10; *t*(13) = −3.24,* p* = 0.006,* d* = 0.95), and these changes reflected a large effect size for working memory training. Participants also improved on measures of inhibitory control. Inhibition Completion Time Scaled Scores (PreTX = 11.2 ± 1.97; MaintTX = 12.07 ± 1.38; *t*(13) = −2.75,* p* = 0.02,* d* = 0.51) and inhibition errors scaled scores (PreTX = 9.21 ± 2.67; MaintTX = 11.29 ± 2.16; *t*(13) = −2.61,* p* = 0.02,* d* = 0.86) improved reflecting medium to large effect sizes for inhibitory control. In addition, parents reported fewer problems with executive functioning at the end of MaintTX (Figure 1(d)) and GEC *T*-Score (PreTX = 61.79 ± 11.36; MaintTX = 56.71 ± 3.63; *t*(13) = 2.14,* p* = 0.05,* d* = 0.40).

### 3.5. Association of Changes in Executive Functioning, SMBG, and HbA1c

Correlations between PreTX-Post MaintTX change scores for measures of executive functioning, SMBG, and HbA1c are provided in Table 2. Improvements in inhibitory control, indexed via participants making fewer errors on the color word interference task (inhibition errors), were associated with increased frequency of SMBG (*r* = 0.78,* p* = 0.001) and decreased HbA1c (*r* = 0.59,* p* = 0.03). Both associations are consistent with large effect sizes. Changes in the other task and parent report measures of executive functioning were not significantly associated with SMBG or HbA1c.

## 4. Discussion

Findings suggest that this web-delivered multicomponent intervention is feasible to deliver in a rural region and might be an effective intervention to increase SMBG, decrease HbA1c, and improve executive functioning in teens with poorly controlled type 1 diabetes. Good compliance with the intervention protocol, evidenced through high rates of completion of therapy sessions and WMT, suggests that this multicomponent intervention was acceptable for the families and provides initial support for the acceptability of utilizing WMT in conjunction with therapy in youth with poorly controlled type 1 diabetes.

More specifically, this intervention showed large effects on SMBG (effect size at the end of ActiveTX for increases in SMBG was 1.56), with teens on average increasing SMBG to over 6 times per day within 3 months' time. Increased SMBG as a result of the intervention may not only help adolescents more effectively manage their daily blood glucose levels, but provide endocrinologists and diabetes educators with sufficient data to assist families in making appropriate changes, if needed, to their insulin dosing. Anecdotally, we found encouraging families to communicate about their newly available blood glucose data with their provider to be important to the treatment process. When compared with available psychosocial interventions on SMBG, which have evidenced small and nonsignificant effect sizes (ES = −0.44–0.13) [2], the current intervention shows promise. In fact, this pilot shows effect sizes similar to intensive multisystemic therapy for diabetes management (ES = 1.09) [32]. With regard to improving HbA1c, this intervention also shows promise. There was a moderate effect size for changes in HbA1c at the end of ActiveTX (*d* = 0.62), which is larger than or similar to the effect sizes for available psychosocial interventions (ES = −0.55–0.59) [1].

The findings from this pilot are also consistent with other intervention studies that have utilized BEI to increase engagement in a health behavior that does not have inherent immediate rewards. Two other pilot trials have utilized BEI to increase SMBG in youth with type 1 diabetes and our findings are consistent with those studies. Stanger et al., 2013, utilized a similar protocol to the BEI intervention piloted here but did not include a maintenance phase of treatment and did not include working memory training [9]. The effect size of that pilot on SMBG (*d* = 1.00) was similar to the large effect size seen in this study. Petry et al., 2015, also piloted a BEI intervention that utilized a different incentive schedule, $.10 per test with bonuses for ≥4 tests with smaller average earnings of $122, and no other counseling or working memory training [14]. The effect size of that pilot on SMBG (*d* = 3.10) is also large; however, only youth testing <4 times per day were recruited into that study, while the current study recruited youth regardless of baseline SMBG testing frequency. Thus, our findings extend prior work on BEI and SMBG, supporting the use of BEI even for youth who are already blood glucose checking >4 times per day but are still experiencing poor glycemic control. These findings support continued research into multicomponent treatments that integrate self-regulatory interventions such as BEI and cognitive training with counseling to facilitate increased SMBG and improved HbA1c.

This pilot provides support for the use of a web-delivery model of a multicomponent family intervention in a rural region, which is important as web-delivery might decrease obstacles to accessing specialized behavioral healthcare (e.g., distance to pediatric endocrinology clinic, few community behavioral health providers with training in teen type 1 diabetes nonadherence). There has been one previous translation of a family-based intervention for teens with poorly controlled type 1 diabetes into a web-delivered format [7]. In those trials, researchers reported similar effect sizes for changes in adherence and glycemic control and similar parent and teen reported working alliance with the therapist in the face-to-face versus web-delivered formats [7, 33]. That particular intervention was delivered from a metropolitan city center and likely reached some families living in rural regions but was not specifically targeting a rural population. The current intervention model delivered in a rural region shows comparatively greater effects on adherence (*d* = 1.56 vs.* d* = 0.45) and slightly greater effects on HbA1c (*d* = 0.62 vs.* d* = 0.40). These interventions provide a promising framework for the delivery of efficacious interventions via the web for youth with poorly controlled type 1 diabetes living in rural regions.

In addition, this is the first trial to our knowledge to utilize cognitive training in youth with type 1 diabetes. Participants evidenced significant improvements in working memory, inhibitory control, and parent reported executive functioning, with large effect sizes for improvements on the digit span task and decreases in errors on the color word interference task. Improvements in inhibition that are not directly trained in the WMT tasks are consistent with a near transfer of executive skill to domains other than working memory [34] and provide further support for the possibly utility in WMT to address executive functioning and self-regulation in youth with poorly controlled type 1 diabetes. Given neuroimaging findings that type 1 diabetes is associated with disrupted brain structure and function [5], interventions targeting cognitive functioning may be particularly important to pursue.

This pilot study suggests multiple directions for future research. Since improvements in SMBG and HbA1c were larger at the end of ActiveTX compared to MaintTX, future iterations of this intervention should focus on strategies for better sustaining the positive change. For example, the duration of ActiveTX could be increased to provide more time for new SMBG habits to develop. Future interventions might also use BEI targeting other self-care behaviors such as carbohydrate counting and insulin dosing that are challenging for teens with poorly controlled type 1 diabetes, or targeting the maintenance of an increasing percentage of blood glucose values in a healthy range. Assessment of other mediators of intervention effects beyond executive functioning, such as self-efficacy, coping skills, psychopathology, and family functioning would also be important. Further, while this study utilized a general working memory training program, some emerging research suggests that domain-specific cognitive training may have a greater effect on modifying executive functioning and self-regulation for specific health behaviors [35]. The development of diabetes-specific cognitive training interventions where the training tasks include stimuli that are associated with diabetes such as meters, insulin injections, pumps, and carbohydrate counting may help to improve the efficacy of cognitive training interventions targeting adherence.

While promising, these preliminary findings require further validation in a larger sample with a randomized control methodology, not only to better assess the intervention effects on SMBG and HbA1c, but also to examine changes in objective metrics of executive functioning where practice effects may be evident. There is also a need for further research examining the cost effectiveness of integrating incentives into our current healthcare delivery models for youth with type 1 diabetes. Given the high cost of hospital admissions for hyper- and hypoglycemic events, as well as the long-term costs associated with vascular disease into adulthood, incentive interventions may reduce overall costs of care. Accordingly, the intervention described here is being evaluated in a randomized control trial, examining efficacy and cost effectiveness.

## Figures and Tables

**Table 1:** Treatment schedule.

	Week	Adolescent	Parent
Active treatment (ActiveTX)	1	Incentives, CBT/MET Session	Incentives, Parent Session
	2	Incentives, CBT/MET Session, WMT	Incentives, Parent Session
	3	Incentives, CBT/MET Session, WMT	Incentives, Parent Session
	4	Incentives, CBT/MET Session, WMT	Incentives, Parent Session
	5	Incentives, CBT/MET Session, WMT	Incentives, Parent Session
	6	Incentives, CBT/MET Session, WMT	Incentives, Parent Session
	7	Incentives, CBT/MET Session	Incentives, Parent Session
	8	Incentives, CBT/MET Session	Incentives, Parent Session
	9	Incentives, CBT/MET Session	Incentives, Parent Session
	10	Incentives, CBT/MET Session	Incentives, Parent Session
	11	Incentives, CBT/MET Session	Incentives, Parent Session

Maintenance treatment (MaintTX)	12		
	13	Incentives, CBT/MET Session	Incentives, Parent Session
	14		
	15		
	16	Incentives, CBT/MET Session	Incentives, Parent Session
	17		
	18		
	19		
	20	Incentives, CBT/MET Session	Incentives, Parent Session
	21		
	22		
	23		
	24		
	25	Incentives, CBT/MET Session	Incentives, Parent Session

**Table 2:** Correlations between change scores for self-monitoring of blood glucose (SMBG), glycemic control (HbA1c), and executive functioning.

	ΔHbA1c	ΔDS	ΔComp	ΔErrors	ΔGEC	MΔ (SD)
ΔSMBG	−0.76^**∗∗**^	−0.21	−0.19	0.78^**∗∗**^	0.04	1.14 (2.01)
ΔHbA1c		0.08	0.15	−0.59^**∗**^	−0.20	0.36 (1.57)
ΔDigit span (DS)			0.19	−0.02	−0.20	2.64 (3.05)
ΔInhibition completion time (comp)				−0.33	−0.05	4.86 (5.78)
ΔInhibition errors (errors)					0.08	1.64 (2.06)
ΔParent report GEC						5.07 (8.88)

^*∗*^ *p* < 0.05; ^*∗∗*^ *p* < 0.01.
